# Associations between Kynurenine pathway metabolites and cognitive dysfunction in major depressive disorder

**DOI:** 10.1371/journal.pone.0328886

**Published:** 2025-08-18

**Authors:** Yingying Pan, Peiwei Xu, Xueli Sun

**Affiliations:** 1 Xiamen Xianyue Hospital, Xianyue Hospital Affiliated with Xiamen Medical College, Fujian Psychiatric Center, Fujian Clinical Research Center for Mental Disorders, Xiamen, China; 2 Mental Health Center, West China Hospital, Sichuan University, Chengdu, China; 3 Department of Psychiatry, The First Affiliated Hospital, Zhejiang University School of Medicine, Hangzhou, China; Donald and Barbara Zucker School of Medicine at Hofstra/Northwell, UNITED STATES OF AMERICA

## Abstract

This research sought to investigate the relationship between cognitive impairment and kynurenine pathway metabolites in individuals diagnosed with major depressive disorder (MDD). A total of 67 patients diagnosed with MDD and 61 healthy controls (HC) were enrolled in this study. Cognitive function was assessed utilizing the MATRICS Consensus Cognitive Battery. Plasma levels of tryptophan (TRP), kynurenine (KYN), kynurenic acid (KYNA), and quinolinic acid (QUIN) were quantified by liquid chromatography-tandem mass spectrometry. Subsequently, we examined the potential associations between metabolites of the KYN pathway and cognitive dysfunction. MDD patients exhibited significantly poorer performance across all cognitive domains, including processing speed, attention/vigilance, working memory, verbal learning, visual learning, reasoning and problem-solving, and social cognition. Inter-group comparisons indicated that levels of KYN, QUIN, and the KYN/TRP ratio in MDD patients were significantly lower than those in HC, whereas KYNA and the KYNA/QUIN ratio were significantly higher. In MDD patients, a negative correlation was observed between KYN levels and working memory (r = −0.302, p = 0.020), and the KYN/TRP ratio was also negatively correlated with working memory (r = −0.307, p = 0.018). Our findings indicate that impaired working memory in MDD is correlated with increased KYN levels and KYN/TRP ratio. This suggests that the KYN pathway may play a role in the pathological mechanisms underlying neurocognitive dysfunction, particularly working memory deficits, in MDD.

## Introduction

Major depressive disorder (MDD) is a chronic and recurrent mental disorder, with a lifetime prevalence estimated between 6.6% and 7.3% [1]. Cognitive symptoms manifest at the onset of MDD [2], and while certain cognitive impairments may ameliorate as mood symptoms subside, approximately one-third of patients continue to endure persistent cognitive deficits following the acute phase [3]. Research on first-degree relatives of MDD patients revealed that a general impairment in cognition is a feature of familial disposition for MDD [4]. Additionally, whole-genome analyses have identified genetic variations associated with cognitive function, including the rs188552424 in TNFRSF21, and rs112979588 in DCAF6 [5]. These findings indicate that cognitive dysfunction is not merely a secondary symptom resulting from mood disturbances, but a core feature of MDD [6]. It may be correlated with a diminished response to treatment, an elevated risk of recurrence, and potential long-term occupational and social dysfunction [7,8]; however, effective treatment options are currently lacking [9].

The pathophysiological mechanisms of cognitive impairment in MDD remain inadequately elucidated. However, current evidence suggests involvement of neural circuitry disruptions, neurotransmitter dysregulation, diminished neuroplasticity, synaptic dysfunction, elevated inflammatory markers, and alterations in the function and reactivity of the hypothalamic-pituitary-adrenal (HPA) axis, among other factors [10–16]. A potential integrative nexus among these hypotheses is the kynurenine (KYN) metabolic pathway [17]. Tryptophan (TRP) metabolism through the KYN pathway is critically modulated by inflammatory and oxidative stress signals. Under normal physiological conditions, hepatic tryptophan 2,3-dioxygenase (TDO) is responsible for maintaining basal KYN metabolism. However, in the context of immune activation, such as during inflammation or stress, there is an induction of extrahepatic indoleamine 2,3-dioxygenase (IDO) across various tissues, including the brain and immune cells, which then becomes the primary rate-limiting enzyme [18].

There exist two divergent perspectives regarding the role of the KYN pathway in cognitive processes. One perspective posits that QUIN exacerbates excitotoxicity by activating the N-methyl-D-aspartate (NMDA) receptor through glutamate stimulation, while KYNA is suggested to exhibit a high affinity for the glycine binding site on the NMDA receptor, thereby exerting a neuroprotective effect by antagonizing the excitotoxicity and necrotic apoptosis mediated by NMDA receptors [19]. On the other hand, elevated levels of KYNA in the brain are implicated in cognitive impairments, particularly those related to deficits in executive function [20]. Furthermore, evidence suggests that KYNA functions as a negative allosteric modulator of the α7 nicotinic acetylcholine receptor (α7nAChR), thereby influencing cognitive processes through its role in cholinergic neurotransmission; however, this assertion remains a subject of ongoing debate [21]. In neurodegenerative conditions such as Down syndrome (DS) and Alzheimer’s disease (AD), alterations in KYN pathway metabolites are significantly associated with cognitive status. Research indicates that individuals with DS who do not exhibit cognitive decline have elevated levels of neuroprotective metabolites, such as KYN and 3-hydroxykynurenine(3-HK). Conversely, increased concentrations of neurotoxic metabolites, including 3-hydroxyanthranilic acid(3-HAA), are observed in both AD and DS patients experiencing cognitive decline. These findings underscore the dysregulation of the KYN pathway as a common mechanistic contributor to the pathogenesis of cognitive impairment [22].

Currently, there is a paucity of research directly investigating the relationship between KYN metabolite levels and cognitive function in patients with MDD, and existing findings are not entirely consistent. Large population cohort studies have demonstrated that plasma levels of KYN and KYNA do not correlate with the severity of depressive or somatic symptoms in individuals with depression; however, these metabolite levels are independently associated with cognitive symptoms [23]. Another study found a negative correlation between serum KYN levels and word learning in female patients, and a negative correlation between serum KYN/TRP ratio and processing speed, word learning, and visual learning, while these correlations were not present in male patients [24]. Compared to MDD, a greater number of cognitive-related studies have been conducted on patients with schizophrenia. These studies have demonstrated a significant correlation between imbalances in neuroprotective and neurotoxic metabolites and cognitive impairment across various dimensions, and this correlation is particularly pronounced in subgroups exhibiting elevated levels of inflammation [25]. A study that concurrently examined patients with schizophrenia and MDD revealed that plasma QUIN levels were specifically associated with cognitive impairment in schizophrenia patients. Notably, this study demonstrated that metabolites such as TRP, KYN, QUIN, and KYNA, among others, were not correlated with cognitive function in individuals with MDD [26]. This finding is inconsistent with previous research conducted on individuals with MDD. Given the extant evidence indicating the involvement of various KYN pathway intermediates in cognitive function, further investigation into the relationship between KYN pathway metabolism and cognitive performance in patients with MDD is warranted. While prior research has established a link between tryptophan metabolites and global cognitive impairment in depression [23,24], substantial gaps in knowledge persist. Firstly, earlier studies have predominantly concentrated on overall cognitive function in MDD, leaving the specific relationship between metabolites and domain-specific cognitive functions, such as working memory, insufficiently investigated. Secondly, although current evidence underscores considerable inconsistency in gender differences concerning tryptophan metabolism [27], there is a paucity of studies that systematically examine gender-stratified relationships between metabolites and cognitive function. Thirdly, most studies predominantly focus on individual metabolites rather than concurrently assessing the integrated KYN/TRP and KYNA/QUIN ratios, which reflect pathway dynamics. This study sought to address these gaps by: (1) conducting domain-specific cognitive assessments using the Measurement and Treatment Research to Improve Cognition in Schizophrenia (MATRICS) Consensus Cognitive Battery (MCCB) [28]; (2) performing a priori sex-stratified analyses to elucidate sexual dimorphism; and (3) simultaneously quantifying four key metabolites (TRP, KYN, KYNA, QUIN) along with their functional ratios. This research has the potential to identify novel targets for interventions aimed at preventing and treating depression, as well as enhancing cognitive performance related to depression.

## Materials and methods

### Subjects

The participants were inpatients at the Department of Psychiatry, West China Hospital, Sichuan University, during the period from 01 November 2021–30 July 2022. Inclusion criteria were as follows: (1) age between 18 and 50 years, with right-handedness; (2) a diagnosis of MDD according to the Structured Clinical Interview for Diagnostic and Statistical Manual of Mental Disorders (5th edition) criteria; (3) a total score of 8 or higher on the 17-item Hamilton Depression Rating Scale (HAMD-17); and (4) the ability to comprehend the study and proficiency in writing and speaking Chinese. The exclusion criteria encompassed the following: (1) a current or historical diagnosis of neurodevelopmental disorder, psychotic disorder, or personality disorder; (2) receipt of electroconvulsive therapy within the past six months; (3) a comorbidity involving current or previous substance abuse or dependence; (4) current or historical physical diseases likely to affect immunological function; (5) medications likely to influence immune function, such as antibiotics or microbiota-altering drugs; and (6) pregnancy or lactation.

Healthy controls (HC) were recruited from the local community through advertising. These individuals were confirmed to be in good health, with no history of psychiatric illness or substance abuse, as determined by the Mini International Neuropsychiatric Interview (version 7.0, MINI 7.0). Participants were excluded if they had a personal history of psychiatric disorders or psychosis, or if such conditions were present among their first-degree relatives. The exclusion criteria for the control group were consistent with those applied to MDD patients.

A total of 67 patients and 61 HC completed the collection of general demographic data, clinical evaluations, cognitive function assessments, and blood sample collection. Before enrolling participants in the study, their informed consent was secured through the signing of a written consent form. The protocol was approved by the Biomedical Ethics Review Committee of West China Hospital, Sichuan University.

### Assessments

The severity of depressive symptoms was assessed using the HAMD-17. To screen for potential bipolar spectrum disorders, the full 32-item Hypomania Checklist (HCL-32) was administered. Additionally, past or current mental disorders were excluded through the MINI 7.0. Cognitive function was qualitatively assessed using the Chinese version of the MCCB [28], encompassing speed of processing, attention/vigilance, working memory, verbal learning, visual learning, reasoning and problem-solving, and social cognition. The clinical validity and test-retest reliability were established for both healthy controls and MDD patients [29,30]. Each domain score was standardized to a T score with a mean of 50 and a standard deviation of 10. For domains encompassing multiple tests, a composite T score was derived by standardizing the mean of the individual T scores.

### Biochemical analysis

All participants underwent an overnight fast, and blood samples were obtained between 8:00 AM and 10:00 AM. The samples were collected in vacutainers without any additional additives and subsequently centrifuged at 4160 rpm at 4°C for 15 minutes within 20 minutes of collection. The supernatant was subsequently aliquoted into centrifuge tubes and promptly frozen at −80°C to prevent repeated freeze-thaw cycles until analysis. To quantify metabolites, plasma proteins were precipitated by the addition of 0.70 mL of 80% methanol, which contained 0.1% ammonia, to 0.10 mL of plasma. The mixture was subjected to vortexing for 2 minutes, followed by ultrasonication at 4°C for 30 minutes. Subsequently, the samples were maintained at −20°C for 1 hour and then centrifuged at 12,000 rpm for 15 minutes at 4°C. The resulting supernatant was filtered through a 0.22-μm membrane prior to analysis.

Plasma TRP, KYN, and KYNA were analyzed using Atlantis Premier BEH Z-HILIC chromatographic columns (1.7 μm, 2.1 × 100 mm), and plasma QUIN was analyzed using an Acquity UPLC HSS T3 chromatographic column (1.8 μm, 2.1 × 100 mm). The ultra-high-speed liquid chromatograph employed, Waters Acquisition UPLC, was procured from Watsch Corporation, USA. The conditions for chromatographic separation include a column temperature of 40°C and a flow rate of 0.450 mL/min. The gradient elution protocol was as follows: 0–1.0 minutes at 100% mobile phase B, 3.0 minutes at 75% mobile phase B, 4.0 minutes at 30% mobile phase B, 5.0 minutes maintained at 30% mobile phase B, and 5.1–6.0 minutes at 100% mobile phase B, with a constant flow rate of 0.450 mL/min. Mobile phase A consisted of 10 mM ammonium formate with 0.1% ammonia in water, while mobile phase B comprised 10 mM ammonium formate with 0.1% ammonia in a mixture of 85% acetonitrile and 15% water. Quantitative analysis was performed using an AB SCIEX 5500 QQQ-MS (SCIEX, USA) equipped with electrospray ionization (ESI) in positive/negative switching mode. The operational parameters included a curtain gas pressure of 35 arb, a collision gas pressure of 7 arb, an ion spray voltage of ±4500 V, a temperature of 450°C, and ion source gas pressures of 55 arb. Compound detection was facilitated by MultiQuant 3.0.2 software (SCIEX), utilizing optimized multiple reaction monitoring (MRM) transitions. Calibration curves, with coefficients of determination (R²) exceeding 0.99, confirmed the linearity of the method.

### Statistical analysis

The demographic disparities between the MDD and HC were assessed utilizing independent sample t-tests and chi-square tests. Analysis of covariance (ANCOVA) was employed to examine the differences in cognitive test scores between the MDD and HC. Considering the sample size (n > 50), the Kolmogorov-Smirnov (K-S) test was employed to evaluate the normality of the distribution of KYN metabolic indicators. The findings indicated that these indicators deviated from a normal distribution. Therefore, the Mann-Whitney U test was employed for between-group comparisons. To thoroughly examine sex-specific effects, we conducted stratified analyses by comparing: (1) female patients diagnosed with MDD to female HC, and (2) male patients with MDD to male HCs. For cognitive domain scores, which adhered to a normal distribution, we employed ANCOVA. In contrast, for metabolite levels that did not conform to a normal distribution, as determined by the Shapiro-Wilk(S-W) test, we utilized the Mann-Whitney U test.

To elucidate the demographic and clinical characteristics influencing KYN metabolic indicators, Spearman correlation analysis was conducted. These identified characteristics were subsequently utilized as control variables in further partial correlation analyses between KYN metabolic indicators and cognitive symptoms. To address the issue of multicollinearity among metabolites while accounting for demographic confounders, we conducted multivariate linear regression analyses utilizing two distinct and mutually exclusive sets of predictors: Set 1 comprised TRP, KYN, KYNA, and QUIN, while Set 2 included the ratios KYN/TRP and KYNA/QUIN. Each predictor set was evaluated against various cognitive domains, with adjustments made for covariates. To ensure the absence of significant multicollinearity, we calculated Variance Inflation Factors (VIF), confirming that all models exhibited VIF values below 3. Additionally, partial correlation analyses between cognitive symptoms and KYN metabolic indicators were performed separately for male and female participants within both the MDD patient cohort and the healthy control group.

All statistical analyses were executed using SPSS Statistics version 26, with p-values less than 0.05 deemed statistically significant.

## Result

### Demographics and clinical characteristics

This study comprised 67 patients diagnosed with MDD and 61 HC. General demographic information and clinical characteristics are presented in Table 1. Statistical analysis revealed no significant differences between the MDD patients and HC in terms of gender, age, education level, tobacco and alcohol use, and body mass index (p > 0.05). Compared to the healthy control group, MDD patients showed significantly impaired performance across multiple cognitive domains, including Speed of Processing, Attention/Vigilance, Working Memory, Verbal Learning, Visual Learning, Reasoning and Problem Solving, and Social Cognition (p < 0.05).

**Table 1:** Participant demographics and clinical characteristics.

	MDD patients (n = 67)	HC (n = 61)	Test statistic	p-Value
Male/female	24/43	29/32	χ2 = 1.808	0.179
Age (years)	28.46 ± 9.88	29.03 ± 9.17	t = 338	0.736
Education (years)	14.07 ± 2.65	14.62 ± 3.43	t = 1.004	0.317
Current smoking	8	4	χ2 = 2.286	0.515
Current drinking	22	23	χ2 = 1.810	0.613
BMI	23.36 ± 4.85	23.21 ± 3.50	t = −0.186	0.853
Age of onset (years)	24.63 ± 9.87	—		
Duration of illness (years)	4.04 ± 4.31	—		
Depressive episode	2(1,4)	—		
Attempted suicides	0(0,1)	—		
HAMD-17	22.50 ± 5.48	—		
Fluoxetine equivalent(mg/d)	30.36 ± 15.51	—		
Speed of processing	44.66 ± 11.21	55.93 ± 7.63	F = 21.691	<0.001
Attention vigilance	43.88 ± 10.20	51.63 ± 7.91	F = 13.829	<0.001
Working memory	39.33 ± 10.04	48.16 ± 7.60	F = 7.160	<0.001
Verbal learning	38.45 ± 9.06	45.97 ± 7.10	F = 9.620	<0.001
Visual learning	47.75 ± 9.34	54.95 ± 6.93	F = 10.407	<0.001
Reasoning and problem solving	47.01 ± 10.36	54.72 ± 10.25	F = 4.706	0.001
Social cognition	39.99 ± 10.53	46.59 ± 9.70	F = 7.623	<0.001
MCCB composite score	39.64 ± 9.72	51.54 ± 6.33	F = 29.206	<0.001

Abbreviations: BMI, Body Mass Index; HAMD, Hamilton Depression Rating Scale; MCCB, MATRICS Consensus Cognitive Battery; MDD, major depressive disorder; HC, healthy controls. Values are presented as mean ± SD unless otherwise specified. Depressive episodes and attempted suicides are presented as median (min, max) due to non-normal distribution.

### Group difference in KYN pathway metabolites

In the combined cohort of patients with MDD and HC, significant correlations were identified between KYN pathway metabolites and various demographic variables. Specifically, plasma KYN levels exhibited a correlation with gender (r = −0.237, p = 0.007), while plasma KYNA levels were associated with gender (r = −0.228, p = 0.010), alcohol consumption (r = 0.183, p = 0.042), and body mass index (BMI) (r = 0.233, p = 0.011). Additionally, the ratio of KYNA/QUIN demonstrated a correlation with alcohol intake (r = 0.181, p = 0.044). In contrast, TRP, QUIN, and the ratio of KYN/TRP did not show significant associations with demographic data. Subsequent group comparisons stratified by gender and alcohol consumption indicated that females exhibited significantly lower plasma KYN levels (Z = −2.673, p = 0.008) and KYNA levels (Z = −2.574, p = 0.010) in comparison to males. Additionally, non-drinkers demonstrated significantly lower plasma KYN levels (Z = −2.126, p = 0.033) and KYNA levels (Z = −2.135, p = 0.033) relative to alcohol drinkers.

In MDD patients, the association between KYN pathway metabolites and a range of clinical variables—including age of onset, disease progression, frequency of depressive episodes, number of suicide attempts, medication dosage (expressed in fluoxetine equivalents), [31] and HAMD scores—was investigated. The analysis revealed a positive correlation between plasma QUIN levels and the number of suicide attempts (r = 0.249, p = 0.042), whereas the KYNA/QUIN ratio exhibited a negative correlation with the number of suicide attempts (r = −0.264, p = 0.031). No significant correlations (p > 0.05) were identified between the other metabolites and the aforementioned clinical variables.

After controlling for age, sex, alcohol and tobacco use, and BMI, the intergroup analysis, as presented in Table 2, patients with MDD exhibited significantly lower levels of KYN (Z = −5.062, p < 0.001), QUIN (Z = −7.46, p < 0.001), and KYN/TRP (Z = −5.269, p < 0.001) compared to HC. Conversely, the level of KYNA (Z = −2.684, p = 0.007) and the KYNA/QUIN (Z = −7.154, p < 0.001) ratio were significantly higher in MDD patients relative to the HC (Table 2).

**Table 2:** Comparison of KYN pathway metabolic measures.

	MDD patients (n = 67)	HC (n = 61)	Z	p-Value
TRP (ng/mL)	4359.68(1448.70)	4044.74(1527.44)	−1.255	0.210
KYN (ng/mL)	236.16(74.12)	293.94(90.45)	−5.062	<0.001
KYNA (ng/mL)	6.56(1.95)	5.97(1.21)	−2.684	0.007
QUIN (ng/mL)	72.77(33.28)	206.95(154.99)	−7.46	<0.001
KYN/TRP (%)	0.05(0.03)	0.07(0.02)	−5.269	<0.001
KYNA/QUIN (%)	0.09(0.06)	0.03(0.05)	−7.154	<0.001

Abbreviations: KYN, kynurenine; TRP, tryptophan; KYNA, kynurenic acid; QUIN, quinolinic acid; MDD, major depressive disorder; HC, healthy controls. Values are presented as median (IQR).

### Sex-stratified comparisons of cognitive function and KYN pathway metabolites

As illustrated in Table 3, the sex-stratified analyses revealed significant differences in cognitive function and KYN pathway metabolites (excluding TRP) between patients with MDD and HC across both genders. An exception was noted in social cognition, where no significant difference was observed between female MDD patients and female HCs. Further analysis within the MDD cohort indicated that female patients exhibited significantly greater cognitive impairment than their male counterparts in several domains: speed of processing (F = 4.605, p = 0.001), attention vigilance (F = 4.981, p < 0.001), working memory (F = 2.588, p = 0.028), and verbal learning (F = 2.879, p = 0.016). Additionally, female MDD patients demonstrated significantly lower levels of KYNA (Z = −2.034, p = 0.042) compared to male MDD patients. No other significant differences in metabolite levels were detected between male and female MDD patients.

**Table 3:** Sex-stratified comparisons of cognitive function and KYN pathway metabolites.

	Male				Female			
	MDD(n = 24)	HC(n = 29)	Test statistic	p-Value	MDD(n = 43)	HC(n = 32)	Test statistic	p-Value
Age (years)	30.67 ± 10.69	28.90 ± 7.06	χ2 = 0.695	0.491	27.23 ± 9.29	29.16 ± 10.84	χ2 = −0.826	0.412
Education (years)	14.12 ± 3.18	15.62 ± 2.80	t = −1.822	0.074	14.05 ± 2.35	13.72 ± 3.74	t = 0.436	0.665
Current smoking	5	3	χ2 = 1.659	0.436	3	1	χ2 = 0.507	0.776
Current drinking	15	18	χ2 = 0.300	0.861	7	5	χ2 = 1.437	0.488
BMI	24.13 ± 4.32	24.36 ± 2.81	t = −0.221	0.826	22.87 ± 5.08	22.08 ± 3.79	t = 0.748	0.457
Speed of processing	49.30 ± 11.79	58.6 ± 7.60	F = 8.385	0.001	42.41 ± 9.57	52.14 ± 6.14	F = 18.409	<0.001
Attention vigilance	48.30 ± 10.03	54.11 ± 7.41	F = 10.180	<0.001	41.39 ± 9.53	49.14 ± 7.73	F = 7.194	0.001
Working memory	41.48 ± 10.54	47.00 ± 8.65	F = 4.662	0.014	38.15 ± 9.85	48.04 ± 6.14	F = 11.480	<0.001
Verbal learning	38.26 ± 7.86	48.29 ± 7.33	F = 13.787	<0.001	38.12 ± 9.11	44.00 ± 6.38	F = 4.834	0.011
Visual learning	49.26 ± 9.75	57.36 ± 5.15	F = 10.295	<0.001	47.39 ± 9.24	53.82 ± 6.85	F = 7.171	0.002
Reasoning and problem solving	48.39 ± 9.99	54.96 ± 9.69	F = 3.610	0.035	46.76 ± 10.43	52.50 ± 10.51	F = 3.235	0.046
Social cognition	42.22 ± 8.79	50.39 ± 8.96	F = 7.116	0.002	38.22 ± 11.24	42.50 ± 9.30	F = 2.087	0.132
MCCB composite score	43.00 ± 9.94	54.43 ± 6.05	F = 23.797	<0.001	37.76 ± 9.19	48.64 ± 5.27	F = 20.177	<0.001
TRP (ng/mL)	4406.92(1645.54)	4044.75(1354.22)	Z = −1.331	0.183	4296.70(1669.16)	4029.00(1803.01)	Z = −.632	0.527
KYN (ng/mL)	248.09(62.81)	295.20(74.11)	Z = −3.377	0.001	227.37(71.60)	290.173(106.77)	Z = −3.503	<0.001
KYNA (ng/mL)	6.98(1.78)	6.17(0.86)	Z = −2.135	0.033	6.50(2.01)	5.61(1.04)	Z = −2.143	0.032
QUIN (ng/mL)	72.51(34.33)	109.69(128.98)	Z = −4.003	<0.001	72.77(31.21)	237.37(107.92)	Z = −6.470	<0.001
KYN/TRP (%)	0.05(0.02)	0.07(0.02)	Z = −4.020	<0.001	0.05(0.03)	0.07(0.02)	Z = −3.353	0.001
KYNA/QUIN (%)	0.10(0.06)	0.06(0.05)	Z = −4.146	<0.001	0.08(0.07)	0.02(0.01)	Z = −6.159	<0.001

Abbreviations: BMI, Body Mass Index; HAMD, Hamilton Depression Rating Scale; MCCB, MATRICS Consensus Cognitive Battery; KYN, kynurenine; TRP, tryptophan; KYNA, kynurenic acid; QUIN, quinolinic acid; MDD, major depressive disorder; HC, healthy controls. MDD, major depressive disorder; HC, healthy controls. Values are presented as mean ± SD unless otherwise specified. KYN pathway metabolites are presented as median (IQR).

### Association between neurocognitive function and levels of KYN pathway metabolites

**Fig 1:**
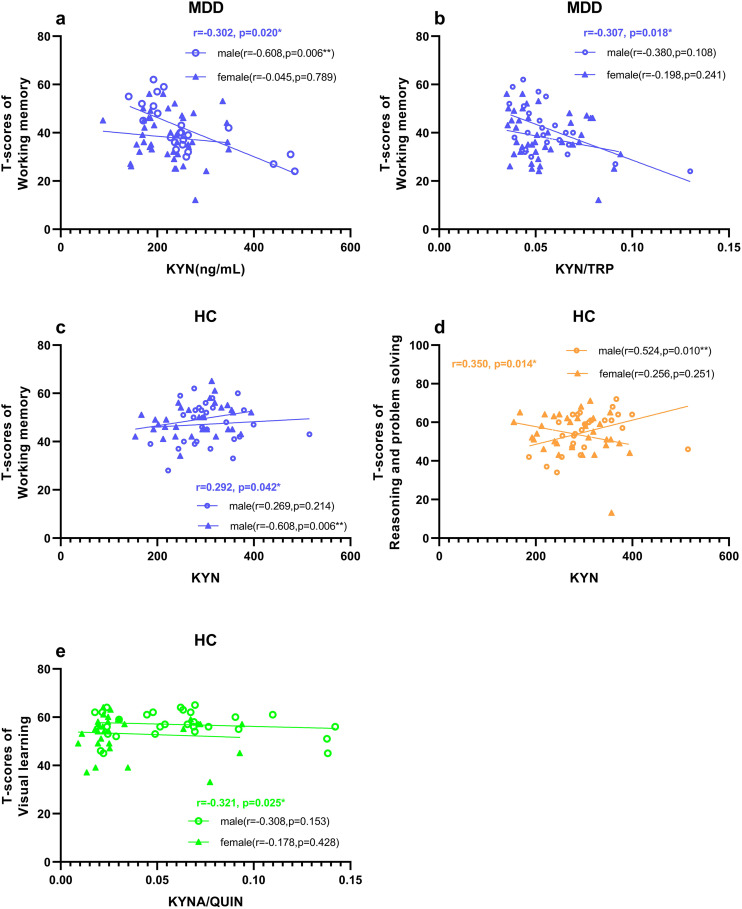
Correlation between KYN pathway metabolites and cognitive function stratified by sex in MDD and HC groups. (a) Plasma KYN levels were negatively correlated with working memory in MDD; (b) Plasma KYN/TRP ratio was negatively correlated with working memory in MDD; (c) Plasma KYN levels were positively correlated with working memory in HC; (d) Plasma KYN levels were positively correlated with reasoning and problem-solving in HC; (e) Plasma KYNA/QUIN ratio was negatively correlated with visual learning in HC. Abbreviations: KYN, kynurenine; TRP, tryptophan; KYNA, kynurenic acid; QUIN, quinolinic acid; MDD, major depressive disorder; HC, healthy controls.

In both groups, we performed correlation analyses between KYN pathway metabolites and various cognitive dimensions. After adjusting for covariates including age, gender, education level, alcohol and tobacco use, and BMI, it was observed that within the MDD group, Fig 1a demonstrates a weak but significant negative correlation between plasma KYN levels and working memory performance in patients with MDD (Fig 1a; r = −0.302, p = 0.020). Additionally, the KYN/TRP ratio showed a weak but significant negative correlation with working memory (Fig 1 b; r = −0.307, p = 0.018). In the HC group, plasma KYN levels exhibited a weak but positive correlation with working memory (Fig 1c; r = 0.292, p = 0.042) and reasoning and problem-solving abilities (Fig 1d; r = 0.350, p = 0.014). Conversely, the plasma KYNA/QUIN ratio demonstrated a weak but negative correlation with visual learning (Fig 1e; r = −0.321, p = 0.025). The multivariate regression analysis, adjusted for demographic confounders, revealed distinct associations between metabolites and cognitive functions. In patients with MDD, KYN (Adjusted R² = 0.190, β = −0.378, p = 0.013) and KYN/TRP (Adjusted R² = 0.189, β = −0.310, p = 0.015) independently predicted impaired working memory. In healthy controls, KYN was positively correlated with reasoning/problem-solving abilities (Adjusted R² = 0.054, β = 0.510, p = 0.014), whereas KYNA/QUIN was negatively associated with visual learning (Adjusted R² = 0.222, β = −0.360, p = 0.026). These findings corroborate our initial conclusions and provide more robust evidence for metabolite-specific cognitive relationships.

Furthermore, sex-stratified correlation analyses demonstrated distinct patterns within the MDD and HC groups (Fig 1). In MDD patients, a significant negative correlation was observed between plasma KYN levels and working memory specifically in males (Fig 1a; r = −0.608, p = 0.006). Conversely, within the HC group, a significant positive correlation between plasma KYN levels and working memory was identified in females (Fig 1c; r = 0.425, p = 0.048), whereas a significant positive correlation between plasma KYN levels and reasoning and problem-solving was found in males (Fig 1d; r = 0.524, p = 0.010), but not in females.

## Discussion

Patients with MDD demonstrated altered KYN pathway metabolism, characterized by decreased production of KYN and QUIN, alongside increased synthesis of KYNA and an elevated KYNA/QUIN ratio. These metabolic shifts were associated with demographic variables such as gender, alcohol consumption, and BMI, as well as clinically significant outcomes. Specifically, lower plasma QUIN levels and a higher KYNA/QUIN ratio were correlated with a reduced incidence of suicide attempts, suggesting a potential neuroprotective role for KYNA. Additionally, the observed inverse relationship between KYN pathway activity, as indicated by the KYN/TRP ratio, and working memory performance suggests that dysregulation in TRP metabolism may contribute to cognitive impairments in MDD.

Decreased plasma KYN levels have notably emerged as the most consistent peripheral biomarker within the KYN metabolic pathway in MDD research, as substantiated by multiple studies [26,32] and corroborated by our findings. However, our study did not identify a significant increase in the conversion rate from TRP to KYN in individuals with MDD. Previous research has suggested that the elevated peripheral KYN/TRP ratio observed in MDD is primarily attributable to TRP depletion rather than an absolute increase in KYN concentration [18]. Notably, this extensive systematic review reveals no evidence of heightened IDO enzyme activity or elevated levels of neurotoxic tryptophan metabolites. In this study, MDD patients showed slightly higher plasma TRP levels than HC, with no significant difference, leading to a notable decrease in the KYN/TRP ratio. The existing literature suggests that antidepressants have a direct inhibitory effect on key enzymes within the KYN pathway. According to Badawy et al., various antidepressants suppress the activity of hepatic TDO, thereby increasing the availability of TRP and reducing the production of KYN [33]. Notably, Maes et al. demonstrated that drug-naïve patients with MDD exhibit elevated activity of IDO, as evidenced by an increased KYN/TRP ratio. Treatment with sertraline was shown to normalize this ratio by concurrently reducing pro-inflammatory cytokines, such as TNF-α and IL-6, and the induction of IDO [34]. In this study, only one of the 67 patients was not receiving antidepressant treatment. This suggests that pharmacotherapy in our cohort may have mitigated inflammation-driven TRP degradation, thereby attenuating the KYN/TRP ratio. An alternative explanation for this phenomenon is the chronic glucocorticoid resistance observed in MDD patients, likely resulting from prolonged elevated cortisol levels, which may lead to decreased TDO activity [35]. This finding is consistent with reports of lower KYN/TRP ratios in patients with recurrent MDD [36]. Moreover, patients entering remission may demonstrate compensatory anti-inflammatory responses, leading to the suppression of IDO and TDO activity [37]. Additionally, dietary factors significantly impact TRP levels, with increased protein intake enhancing TRP availability and carbohydrate consumption affecting free TRP concentrations [38,39]. Despite the collection of fasting blood samples to mitigate the influence of recent food intake, this study did not examine the participants’ habitual dietary patterns. In conclusion, the observed reduction in the KYN/TRP ratio is likely indicative of a combination of treatment-induced enzyme suppression, glucocorticoid receptor resistance in chronic disease, and anti-inflammatory conditions—factors that overshadow the transient effects of TRP depletion in major depressive disorder. Future research should stratify participants based on medication history, illness chronicity, and inflammatory biomarkers (such as CRP and IL-6) to further elucidate these dynamics.

We investigated peripheral alterations in these downstream metabolites of KYN. In alignment with prior research, our findings indicated a reduction in peripheral plasma QUIN levels within the MDD patient cohort [40]. However, the association between QUIN and MDD is intricate, as various studies have documented either no change or an increase in QUIN levels in MDD [41]. Our findings of decreased QUIN levels concomitant with elevated KYNA diverge from previous research on severe MDD subtypes, such as those with melancholic or psychotic features, which have documented increased neurotoxic QUIN levels [41]. This discrepancy may suggest a compensatory neuroprotective mechanism within our cohort, aligning with evidence that anti-inflammatory conditions inhibit QUIN synthesis [37]. These results may imply that, within the context of MDD, there is an adaptive shift towards neuroprotection, potentially influenced by pharmacological intervention. Recent evidence highlights that the kynurenine pathway operates through a delicate balance between neurotoxic (e.g., QUIN, 3HK) and neuroprotective metabolites (e.g., KYNA). In depression, this equilibrium is disrupted by immune-inflammatory processes, resulting in increased neurotoxic potential that contributes to neurodegeneration and cognitive deficits [42]. Our findings of heightened KYNA/QUIN ratio—a marker of reduced neurotoxicity—align with this framework. Correlation analyses with clinical features indicated that elevated plasma QUIN levels, as well as the KYNA/QUIN ratio, were associated with an increased number of suicide attempts, yet showed no significant relationship with HAMD-17 scores. These findings are consistent with prior research indicating that the peripheral imbalance of KYNA and QUIN in MDD is not correlated with depressive symptoms, but is independently associated with suicidal ideation [43–45]. The observed positive correlation between QUIN and suicide attempts underscores the significance of neurotoxic tryptophan catabolites in the pathophysiology of suicidal behavior. Elevated levels of QUIN NMDA receptor-mediated excitotoxicity and oxidative stress, leading to compromised hippocampal integrity and impaired emotional regulation [42]. In contrast, a higher KYNA to QUIN ratio, which is neuroprotective, may alleviate these detrimental effects, thereby highlighting its potential utility as a biomarker for stratifying suicide risk.

To mitigate the influence of confounding variables, we performed an analysis examining the correlation between KYN pathway metabolite levels and demographic factors, including gender and alcohol consumption. This study demonstrates that gender significantly influences KYN metabolites, with females exhibiting lower plasma levels of KYN and KYNA compared to males. These findings align with prior research and suggest that the interaction between sex hormones and the immune system may underlie these differences [27]. Additionally, alcohol consumption appears to disrupt KYN metabolism by modulating liver TDO activity [46]. In subsequent analyses, we will account for these demographic and clinical variables as covariates to mitigate potential confounding effects on KYN metabolite levels.

The principal finding of this study was that elevated plasma KYN levels and an increased KYN/TRP ratio were associated with impaired working memory in individuals with MDD. This finding aligns with prior research that has identified a relationship between KYN metabolism and cognitive function [24,47–49]. In rodent models, transient exposure to KYN during early life has been shown to partially affect working memory and attention in adulthood [50]. Additionally, acute elevation of brain KYN levels in adulthood has been demonstrated to induce recognition memory impairment [51], while inhibition of IDO activity has been found to enhance memory function [52]. In human studies, serum KYN levels and the KYN/TRP ratio have been specifically associated with learning and processing speed in female patients with MDD [24]. Among elderly MDD patients, variations in KYN metabolism have been observed between those with and without memory impairment, with a trend indicating that the serum KYN/TRP ratio may predict memory impairment [49]. Intervention studies utilizing probiotics have demonstrated that a reduction in plasma KYN concentration following treatment may contribute to improvements in attention, word learning, and other cognitive functions in individuals with MDD [53]. These studies elucidate the association between peripheral and central KYN pathway activation and cognitive impairment; however, the precise mechanisms underlying this relationship remain to be determined. Given the correlation between KYN and working memory and attention, modulating the KYN pathway to enhance cognitive performance in MDD presents a promising target for psychopharmacological interventions. This approach could potentially contribute to more effective clinical management of MDD. Although the observed correlations between KYN pathway metabolites and cognitive domains reached statistical significance, the correlation coefficients (r < 0.4) suggest weak associations. These findings should be regarded as preliminary evidence of biological associations rather than definitive predictors. Future longitudinal studies involving larger cohorts are necessary to clarify the clinical significance of these subtle relationships.

This research delineated a gender-specific pattern in the association between cognitive dysfunction and KYN pathway metabolites in individuals with MDD. The findings indicated that females presented with lower baseline levels of KYN and KYNA compared to males, which may be attributed to the interactions between sex hormones and immune pathways [42]. This phenomenon could potentially contribute to the more pronounced cognitive impairments observed in female MDD patients within our study, as fluctuations in estrogen levels may exacerbate inflammation-induced IDO activation and the production of neurotoxic tryptophan catabolites. In contrast, male patients exhibited a stronger correlation between elevated KYN levels and deficits in working memory. These findings suggest distinct neurotoxic and neuroprotective balances influenced by sex hormones. The results indicate that enhancing neuroprotective pathways, such as through the use of kynurenine monooxygenase (KMO) inhibitors or anti-inflammatory treatments, may be more effective for female patients. Conversely, male patients may benefit from interventions targeting KYN-mediated excitotoxicity, such as NMDA receptor modulators. These insights underscore the necessity for tailored therapeutic strategies to optimize cognitive outcomes in individuals with MDD.

## Limitations

Several limitations must be taken into account when interpreting these findings. Firstly, the ability to thoroughly understand the causal relationships between cognitive performance and KYN pathway metabolism is limited by the cross-sectional design of this study and the restricted metabolite profiling. This limitation precludes the analysis of other biologically significant intermediates, such as 3-HK, xanthurenic acid (XA), and anthranilic acid (AA). Secondly, the study did not quantify several relevant biological variables, including inflammatory cytokines (e.g., IL-6, IFN-γ), oxidative stress biomarkers, or the activity of rate-limiting enzymes such as IDO. As a result, we were unable to investigate direct pathophysiological correlations between kynurenine pathway dysregulation and clinical symptoms. Future research incorporating these biomarkers would more effectively elucidate the kynurenine pathway-mediated mechanisms underlying cognitive and clinical symptoms in MDD. Third, KYN pathway metabolites were measured in peripheral blood rather than cerebrospinal fluid (CSF). It is crucial to recognize that there are significant differences in tryptophan catabolite profiles between central and peripheral compartments. We acknowledge that peripheral measurements, particularly those obtained from plasma, may not accurately represent central neurochemical activity. This discrepancy is due to factors such as the compartmentalized regulation of pathways, the dynamics of the blood-brain barrier, and methodological variability in sample processing [54]. Future research should prioritize direct assessments of central nervous system (CNS) activity or seek to validate serum-based proxies for central IDO and kynurenine monooxygenase (KMO) activity. Additionally, although fasting blood samples were collected to mitigate the effects of recent food intake, we did not evaluate the participants’ habitual dietary patterns. This limitation hinders our ability to analyze the potential dietary influences on metabolite levels, which could serve as a significant confounding factor. Future research should prioritize the prospective monitoring of dietary intake in conjunction with metabolite measurements. Finally, the relatively limited sample size may constrain the generalizability of our findings and reduce statistical power to detect smaller effect sizes, especially in subgroup analyses such as gender-stratified or medication-specific effects. Consequently, future large-scale studies are warranted to validate these results.

## Conclusion

Our findings indicate that impaired working memory in MDD is correlated with elevated levels of KYN and an increased KYN/TRP ratio. This suggests that the KYN pathway may play a role in the pathological mechanisms underlying neurocognitive dysfunction, particularly working memory deficits, in MDD. Consequently, the KYN pathway could be considered a novel target for pharmacological intervention, offering a promising strategy for the prevention and treatment of depression, as well as for enhancing cognitive performance associated with the disorder.

## Supporting information

S1 Data Minimal dataset. (XLSX)
